# Implication of miR-155-5p and miR-143-3p in the Vascular Insulin Resistance and Instability of Human and Experimental Atherosclerotic Plaque

**DOI:** 10.3390/ijms231810253

**Published:** 2022-09-06

**Authors:** Paula González-López, Carla Ares-Carral, Andrea R. López-Pastor, Jorge Infante-Menéndez, Tamara González Illaness, Melina Vega de Ceniga, Leticia Esparza, Nuria Beneit, José Luis Martín-Ventura, Óscar Escribano, Almudena Gómez-Hernández

**Affiliations:** 1 Hepatic and Vascular Diseases Laboratory, Biochemistry and Molecular Biology Department, School of Pharmacy, Complutense University of Madrid, 28040 Madrid, Spain; 2 Department of Angiology and Vascular Surgery, Hospital de Galdakao-Usansolo, 48960 Galdakao, Spain; 3 Biocruces Bizkaia Health Research Institute, 48903 Barakaldo, Spain; 4 IIS-Fundation Jimenez-Diaz, Autonoma University of Madrid, 28040 Madrid, Spain; 5 Centro de Investigación Biomédica en Red de Enfermedades Cardiovasculares (CIBERCV), 28029 Madrid, Spain

**Keywords:** atherosclerosis, miR-155-5p, miR-143-3p, unstable plaque, apoptosis, AKT, IGF-IIR

## Abstract

(1) Background: Cardiovascular diseases (CVDs) are the main cause of death in developed countries, being atherosclerosis, a recurring process underlying their apparition. MicroRNAs (miRNAs) modulate the expression of their targets and have emerged as key players in CVDs; (2) Methods: 18 miRNAs were selected (Pubmed and GEO database) for their possible role in promoting atherosclerosis and were analysed by RT-qPCR in the aorta from apolipoprotein E-deficient (*ApoE*^−/−^) mice. Afterwards, the altered miRNAs in the aorta from 18 weeks-*ApoE*^−/−^ mice were studied in human aortic and carotid samples; (3) Results: miR-155-5p was overexpressed and miR-143-3p was downregulated in mouse and human atherosclerotic lesions. In addition, a significant decrease in protein kinase B (AKT), target of miR-155-5p, and an increase in insulin-like growth factor type II receptor (IGF-IIR), target of miR-143-3p, were noted in aortic roots from *ApoE*^−/−^ mice and in carotid plaques from patients with advanced carotid atherosclerosis (ACA). Finally, the overexpression of miR-155-5p reduced AKT levels and its phosphorylation in vascular smooth muscle cells, while miR-143-3p overexpression decreased IGF-IIR reducing apoptosis in vascular cells; (4) Conclusions: Our results suggest that miR-155-5p and miR-143-3p may be implicated in insulin resistance and plaque instability by the modulation of their targets AKT and IGF-IIR, contributing to the progression of atherosclerosis.

## 1. Introduction

Cardiovascular diseases are the main cause of premature death in developed countries and usually progress with an asymptomatic period [1]. Atherosclerosis is usually the underlying cause of cardiovascular diseases like coronary artery disease or myocardial infarction [2,3,4].

Insulin-resistant states are associated with metabolic abnormalities that include glucotoxicity, lipotoxicity and inflammation, and which also lead to endothelial dysfunction. Therefore, hyperglycaemia, hyperlipidemia and proinflammatory cytokines are known to selectively impair the phosphoinositide 3-kinase (PI3K)/AKT/ endothelial nitric oxide synthase (eNOS) pathway, increase oxidative stress and enhance the release of endothelin 1 (ET-1) from the endothelium [5]. Different manifestations associated with insulin resistance, including dyslipidemia, hyperglycaemia, inflammation and obesity, may be intermediary mediators along with insulin resistance to generate endothelial dysfunction [6,7].

MicroRNAs (miRNA) are small non-coding RNAs conserved between species that regulate the expression of genes. Since their discovery, hundreds of these transcripts have been described unravelling the role they play in development or disease [8]. In this sense, both inhibition and overexpression of miRNAs that participate in the establishment and progression of CVDs have been described. For instance, miR-145 and miR-143 regulate vascular smooth muscle cell (VSMC) fate and plasticity [9], whereas miR-24-3p has been described as an important regulator in VSMCs proliferation and apoptosis [10]. Moreover, miR-27b, -130a and -210, which are involved in the maintenance of endothelial cell homeostasis, are increased in patients with peripheral arterial disease [11] and downregulated in T2DM patients after treatment with liraglutide [12].

The effect of miR-155-5p in several diseases is controversial. Some studies demonstrated that this miRNA protects against the progression of diseases such as cervical cancer by targeting phosphoinositide-dependent protein kinase 1 (PDK1) [13] or type-2-diabetes by targeting the transcription factor MafB (Mafb) [14]. During atherosclerosis progression, miR-155-5p overexpression promotes inflammation [15] and oxidized low-density lipoprotein uptake in macrophages [16].

The miR-143-3p is well studied in the progress and development of different types of cancer like adenocarcinoma [17] or ovarian cancer [18]. In recent years, miR-143-3p has emerged as a possible regulator of myocardial infarction [19,20]. In contrast, the role of this miRNA plays in atherosclerosis remains mostly unknown. 

For these reasons, 18 miRNAs were selected by searching in PubMed and GEO database for their possible role in promoting atherosclerosis and analyzed by RT-qPCR in the aorta from *ApoE^−/−^* and wild-type mice. To carry out this objective, we have used a classic experimental model of atherosclerosis, *ApoE^−/−^* mice under a standard diet (STD) or a high-fat diet (HFD) for 8 and 18 weeks. Then, the altered miRNAs in the aorta from *ApoE^−/−^* mice fed with the corresponding diets for 18 weeks were studied in human aortic and carotid samples, using vascular samples from control subjects (CAs), subjects with fibrolipidic plaques (FAs) or patients with advanced carotid atherosclerosis (ACA). Finally, we focused on the role of miR-155-5p and miR-143-3p and their targets (AKT and IGF-IIR, respectively) in the progression of experimental and human atherosclerosis, as well as its implication in vascular insulin resistance. To unravel the molecular mechanism by which these miRNAs promote atherosclerosis, we performed in vitro overexpression experiments in human vein endothelial cells (HUVECs) and vascular smooth muscle cells (VSMCs).

## 2. Results

### 2.1. miR-143-3p and miR-155-5p Levels Are Altered in Aorta from ApoE^−/−^ Mice

*ApoE^−/−^* mice under HFD were used as an animal model of atherosclerosis. Firstly, we confirmed that *ApoE^−/−^* mice had a significant increase in body weight and weight gain in addition to hypercholesterolemia and hypertriglyceridemia (Supplemental Table S1), being significantly higher in *ApoE^−/−^* fed with HFD for 18 weeks. 

**Figure 1:**
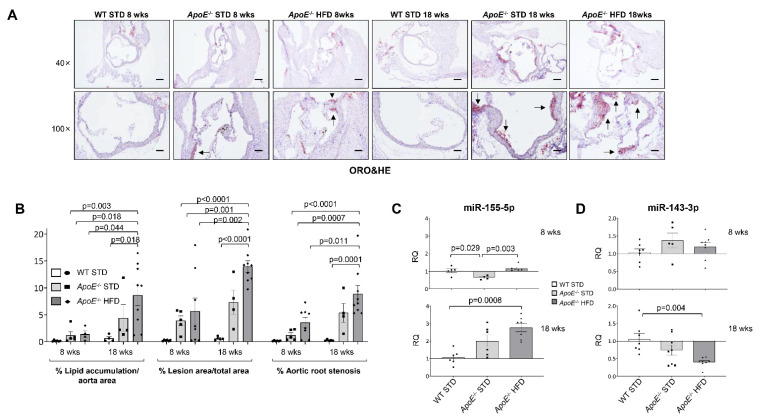
miR-155-5p and miR-143-3p expression in the aorta of the mouse model of atherosclerosis. (**A**) Representative images of the Oil Red O staining from the aortic roots of the six groups of experimental mouse model of atherosclerosis. Magnification 40× (scale bar = 200 µm); magnification 100× (scale bar = 100 µm). The black arrows point out the lipid depots in the aortic roots. (**B**) Quantification of the percentage of lipid accumulation, lesion area and stenosis of aortic root from the mouse model. WT STD 8 wks (*n* = 6); *ApoE^−/−^* STD 8 wks (*n* = 5); *ApoE^−/−^* HFD 8 wks (*n* = 4); WT STD 18 wks (*n* = 4); *ApoE^−/−^* STD 18 wks (*n* = 4); *ApoE^−/−^* HFD 18 wks (*n* = 9). Relative expression of miR-155-5p (**C**) and miR-143-3p (**D**) in the aorta of the three experimental groups at 8 (**upper** graphics) and 18 weeks (**lower** graphics) of diet was measured by qPCR. Amplification of miR-191-5p was used in the same reaction of all samples as an internal control. WT = Wild type group; STD = standard type diet; *ApoE^−/−^* = *ApoE* deficient mice; HFD = high-fat diet; wks = weeks. qPCR miR-155-5p: WT STD 8 wks (*n* = 5); *ApoE^−/−^* STD 8 wks (*n* = 4); *ApoE^−/−^* HFD 8 wks (*n* = 6); WT STD 18 wks (*n* = 6); *ApoE^−/−^* STD 18 wks (*n* = 6); *ApoE^−/−^* HFD 18 wks (*n* = 7). qPCR miR-143-3p: WT STD 8 wks (*n* = 7); *ApoE^−/−^* STD 8 wks (*n* = 5); *ApoE^−/−^* HFD 8 wks (*n* = 8); WT STD 18 wks (*n* = 7); *ApoE^−/−^* STD 18 wks (*n* = 8); *ApoE^−/−^* HFD 18 wks (*n* = 8).

After that, we analysed vascular damage by Oil Red O (ORO) staining of aortic roots (Figure 1A). A significant increase in lipid depot, lesion area and % of stenosis was noted in aortic roots from *ApoE^−/−^* HFD 18 wks in comparison with the other groups (Figure 1B). Similarly, *en face* analysis of ORO-stained whole aorta showed a significant increase in lesion area in *ApoE^−/−^* under HFD for 18 weeks vs. Control STD and *ApoE^−/−^* STD of the same age (Supplementary Figure S1).

PubMed and GEO Databases were used to perform a search of miRNAs that could play a role in atherosclerosis. After the search, a screening of the selected miRNAs was performed in the aorta of all groups of mice (Supplemental Figure S2 and Table S2). We observed that miR-155-5p was significantly overexpressed in *ApoE^−/−^* mice after 18 weeks of HFD compared with WT STD mice (Figure 1C); while miR-143-3p was significantly downregulated in *ApoE^−/−^* mice after 18 weeks of HFD vs. WT mice (Figure 1D).

Moreover, we have established correlations between miR-155-5p or miR-143-3p and atherosclerosis progression (% lipid accumulation/aorta area, % lesion area/total area or % aortic root stenosis) showed that miR-155-5p levels significantly and positively correlate with % stenosis and lesion area (Supplemental Figure S3A,B, respectively). On the other hand, we found a significant and inverse correlation between miR-143-3p levels and % lipid accumulation or lesion area (Supplemental Figure S3C,D, respectively). 

### 2.2. miR-143-3p and miR-155-5p Levels Are Also Altered in Human Atherosclerotic Carotid Plaque

**Figure 2:**
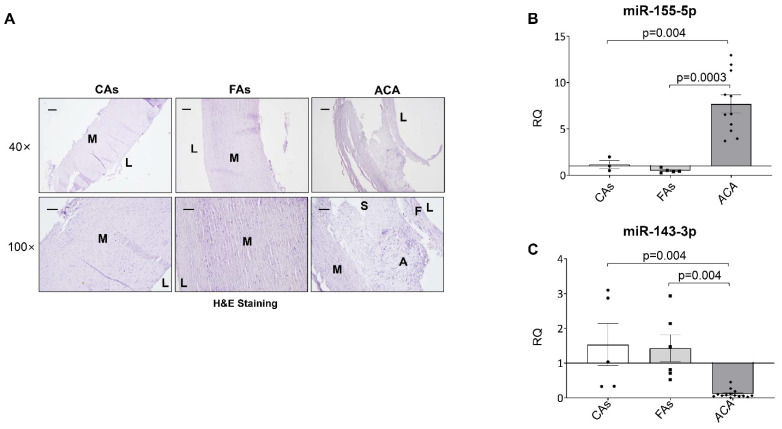
Characterization of the atherosclerotic plaques and miRNA expression of the human vascular samples. (**A**) Representative images of the Hematoxylin and Eosin staining from the aorta of control subjects (CAs) and subjects with fibrolipidic lesions (FAs) and the carotid artery from ACA patients (ACA). Magnification 40× (scale bar = 200 µm) and 100× (scale bar = 100 µm). Relative quantification of the levels of the miR-155-5p (**B**) and miR-143-3p (**C**) by qPCR. FAs= fibrolipidic plaque; ACA = advanced carotid atherosclerotic plaque; M = media; F = fibrous; S = shoulder; A = atheroma; L = lumen. qPCR miR-155-5p: Control subjects (*n* = 3); FAs (*n* = 5); ACA patients (*n* = 11). qPCR miR-143-3p: Control subjects (*n* = 5); fibrolipidic patients (*n* = 6); ACA patients (*n* = 14).

To confirm whether the levels of the studied miRNAs in the murine model of atherosclerosis might have a clinical relevance in human atherosclerosis, we analyzed the levels of different miRNAs in vascular samples from control subjects (CAs), subjects with fibrolipidic plaque (FAs) and patients with human advanced atherosclerosis undergoing carotid endarterectomy (ACA) (Supplemental Table S2). Firstly, we performed H&E stainings, and we could differentiate regions as media in samples from CAs and FAs and media, fibrous and shoulders in samples from ACA (Figure 2A). Histological analysis revealed that complicated plaques from ACA contained an intraplaque hemorrhage and/or a certain degree of calcification with a higher percentage of inflammatory cells. The adjacent non-complicated regions were composed of fibrous thickening with a variable content of VSMCs (Figure 2A).

In serial sections of samples used for histological characterization, we isolated miRNAs and analyzed the levels of miR-155-5p (Figure 2B) and miR-143-3p (Figure 2C) by qPCR, showing that miR-155-5p expression was significantly upregulated (Figure 2B) and miR-143-3p downregulated (Figure 2C) in the ACA patients compared with the other two groups.

### 2.3. AKT Expression Is Modulated by miR-155-5p Levels in Atherosclerosis and Non-Alcoholic Fatty Liver Disease

**Figure 3:**
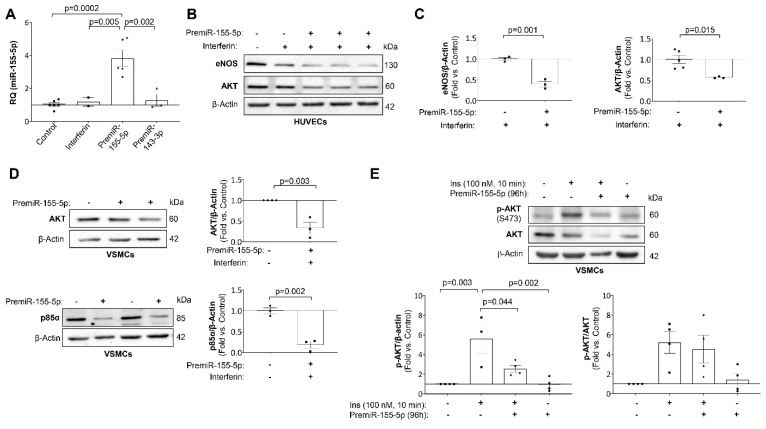
miR-155-5p reduced AKT expression and its activation in vascular cells. (**A**) Relative quantification of the expression levels of miR-155-5p in transfected HUVECs. (**B**) Representative Western blot images of AKT and eNOS expression and (**C**) their quantification in transfected HUVECs. (**D**) Representative Western blot images of the expression of p85α and AKT (**left**) and their quantification (**right**) in transfected HUVECs. (**E**) Representative Western blot images of AKT phosphorylation in response to insulin stimulation in transfected VSMCs (**upper**) and their quantification (**lower**). HUVECs = human umbilical vein endothelial cells; VSMCs = vascular smooth muscle cells; eNOS = endothelial nitric oxide synthase; AKT = protein kinase B; p85α= phosphoinositide-3-kinase regulatory subunit 1; Ins = insulin. All the in vitro experiments were performed in triplicate.

To study the effect of miR-155-5p overexpression in vascular insulin resistance, HUVECs and VSMCs were transfected with a specific precursor for miR-155-5p. After 72 h of transfection, miR-155-5p was overexpressed in both vascular cell lines (Figure 3A). Following an extensive search in different databases, AKT, eNOS and p85α were the most relevant predicted miR-155-5p targets in atherosclerosis (Supplemental Figure S4A,B).

**Figure 4:**
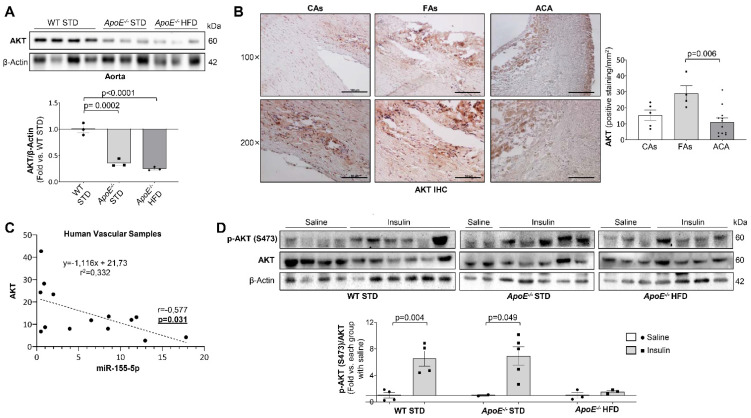
AKT levels are downregulated in the human samples and the mouse model where its activity is also impaired. (**A**) Representative Western blot images of the expression of AKT (**upper**) and their quantification (**lower**) in murine aorta samples. (**B**) Representative images of AKT levels measured by immunohistochemistry in the aorta and carotid human samples (**left**) and their quantification (**right**) expressed as % positive staining/mm^2^. Magnification 100× (scale bar = 100 µm); magnification 200× (scale bar = 50 µm). (**C**) Scatter plot and Spearman’s r correlation between the expression of miR-155-5p and AKT expression in human samples. (**D**) Representative Western Blot images of AKT phosphorylation (**upper**) and their quantification (**lower**) in aortas from mice subjected to in vivo insulin signaling studies. WT = Wild type group; STD = standard type diet; *ApoE^−/−^* = *ApoE* deficient mice; HFD = high fat diet; AKT = protein kinase B; FAs = fibrolipidic plaque; ACA = advanced carotid atherosclerotic plaque. Mouse model AKT Western blot: WT STD 18 wks (*n* = 3); *ApoE^−/−^* STD 18 wks (*n* = 3); *ApoE^−/−^* HFD 18 wks (*n* = 3); AKT immunohistochemistry: Control subjects (*n* = 5); FAs (*n* = 4); ACA (*n* = 12). miR-155-5p-AKT correlation (*n* = 14). In vivo signalling experiments: WT STD 18 wks saline (*n* = 4); WT STD 18 wks insulin (*n* = 4); *ApoE^−/−^* STD 18 wks saline (*n* = 2); *ApoE^−/−^* STD 18 wks insulin (*n* = 5); *ApoE^−/−^* HFD 18 wks saline (*n* = 3); *ApoE^−/−^* HFD 18 wks insulin (*n* = 3).

After 96 h of transfection with the miR-155-5p precursor, eNOS and AKT were significantly downregulated in HUVECs (Figure 3B,C). Similarly, the expression of AKT and p85α significantly decreased in transfected VSMCs (Figure 3D). Furthermore, AKT expression was also significantly downregulated in the aorta of the 18 weeks-fed *ApoE^−/−^* mice (Figure 4A) and in the carotid from patients with advanced carotid atherosclerosis (Figure 4B). Moreover, in the carotid of patients with atherosclerosis, miR-155-5p levels negatively correlated with the expression of its target, AKT (Figure 4C).

Next, we assessed whether the downregulation of AKT induced by miR-155-5p could impair its phosphorylation. In fact, VSMCs overexpressing miR-155-5p showed a significant reduction in AKT phosphorylation induced by 100 nM insulin for 10 min (Figure 3E). Regarding in vivo insulin signaling, insulin administration did not induce AKT phosphorylation in the aorta from *ApoE^−/−^* HFD 18 wks, which had high levels of miR-155-5p. Conversely, it induced a significant increase in AKT phosphorylation in WT STD and *ApoE^−/−^* STD mice (Figure 4D).

**Figure 5:**
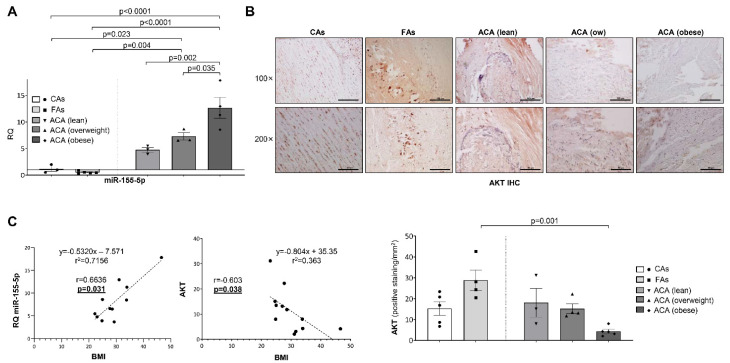
miR-155-5p and AKT expression correlated with the body mass index in human samples. (**A**) Expression levels of miR-155-5p in human carotid samples of patients stratified according to the Body Mass Index criteria of the World Health Organization. (**B**) Representative images of AKT immunohistochemistry (**upper**) and their quantification (**lower**) in human samples of patients stratified according to the Body Mass Index criteria of the World Health Organization. Magnification 100× (scale bar = 100 µm); magnification 200× (scale bar = 50 µm). (**C**) Scatter plots and Spearman’s r correlations between the Body Mass Index and miR-155-5p expression (**left**) or AKT expression (**right**). AKT = protein kinase B; FAs = fibrolipidic plaque; ACA = advanced carotid atherosclerotic plaque; ow = overweight; IHC = immunohistochemistry; BMI = body mass index. miR-155-5p qPCR: CAs (*n* = 3); FAs (*n* = 5); ACA (lean) (*n* = 3); ACA (overweight) (*n* = 3); ACA (obese) (*n* = 4). AKT immunohistochemistry: CAs (*n* = 5); FAs (*n* = 4); ACA (lean) (*n* = 3); ACA (overweight) (*n* = 4); ACA (obese) (*n* = 5). Correlation between miR-155-5p and BMI (*n* = 11) and between AKT and BMI (*n* = 12).

Moreover, when patients were classified by their body mass index (BMI) following the criteria established by the World Health Organization (lean, overweight and obese), we observed a significant increase in miR-155-5p expression (Figure 5A), as well as a progressive decrease in AKT (Figure 5B), as the BMI increased. This was supported by a significant and positive correlation between the BMI and both miR-155-5p (Figure 5C, left) and a significant and inverse correlation between the BMI and AKT levels (Figure 5C, right). In addition, we also separated the patients into diabetic and non-diabetic, which revealed a high and significant increase in miR-155-5p in diabetic patients with ACA (Supplemental Figure S5). 

**Figure 6:**
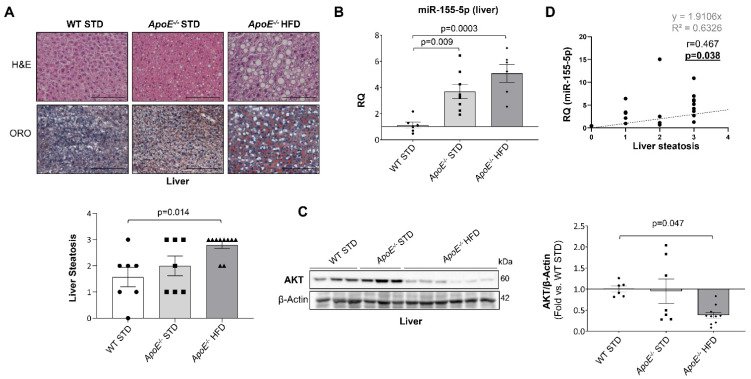
miR-155-5p is overexpressed and AKT downregulated in the liver of the mouse model after 18 weeks of high-fat diet. miR-155-5p is overexpressed and AKT downregulated in the liver of the mouse model after 18 weeks of high-fat diet. (**A**) Representative images of H&E staining to perform liver histological analysis (**upper**) and of Oil Red O staining (**lower**) to evaluate hepatic-specific lipid content. Magnification 200× (scale bar = 25 µm). Below, its corresponding graph that show the quantification of liver steatosis. (**B**) Relative expression of miR-155-5p in murine liver samples measured by qPCR. (**C**) Representative Western blot images of AKT expression (**left**) and their quantification (**right**) in liver samples from the mouse groups. (**D**) Scatter plots and Spearman’s r correlations between liver steatosis and hepatic miR-155-5p expression. WT= Wild type group; STD = standard type diet; *ApoE^−/−^* = *ApoE* deficient mice; HFD = high-fat diet; AKT = protein kinase B. Image and its quantification steatosis liver: WT STD 18 wks (*n* = 7), *ApoE^−/−^* STD 18 wks (*n* = 7) and *ApoE^−/−^* HFD (*n* = 10) mice after 18 weeks on the diet. miR-155-5p qPCR liver: WT STD 18 wks (*n* = 6); *ApoE^−/−^* STD 18 wks (*n* = 8); *ApoE^−/−^* HFD 18 wks (*n* = 6). Western blot of AKT liver: WT STD 18 wks (*n* = 6); *ApoE^−/−^* STD 18 wks (*n* = 7); *ApoE^−/−^* HFD 18 wks (*n* = 10).

Since the *ApoE^−/−^* mice also showed hepatic steatosis after 18 weeks of diet (Figure 6A), the expression of miR-155-5p was analysed in the liver by qPCR. Hepatic expression of miR-155-5p was significantly higher in both *ApoE^−/−^* mice groups (Figure 6B), which correlated with a downregulation in liver AKT expression in *ApoE^−/−^* HFD mice (Figure 6C). Moreover, a significant and positive correlation was observed between miR-155-5p hepatic levels and hepatic steatosis (Figure 6D).

### 2.4. IGF-IIR Expression Is Modulated by miR-143-3p

**Figure 7:**
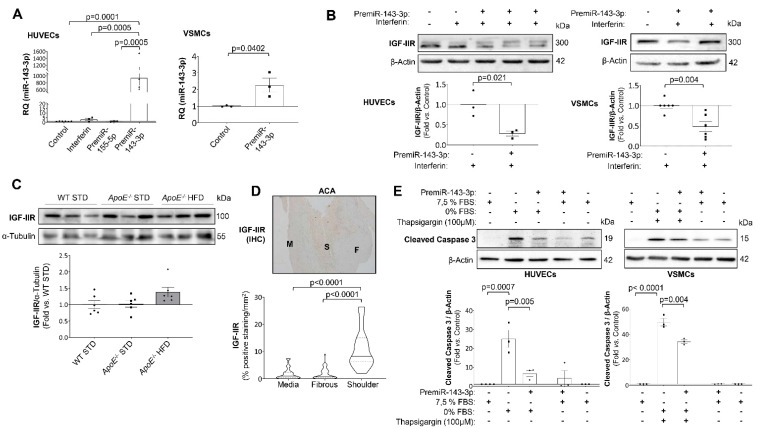
miR-143-3p protects against apoptosis in endothelial and vascular smooth muscle cells by targeting IGF-IIR. (**A**) Relative quantification of the expression levels of miR-143-3p in transfected HUVECs (**left**) and VSMCs (**right**). (**B**) Representative Western blot images of IGF-IIR expression (**upper**) and their quantification (**lower**) in transfected HUVECs (**left** panels) and VSMCs (**right** panels). (**C**) Representative Western blot images of IGF-IIR expression (**upper**) and their quantification (**lower**) in murine aorta samples. (**D**) Representative immunohistochemical analysis of IGF-IIR expression in advanced carotid atherosclerosis patients (**upper**) and its quantification (**lower**) expressed in %positive staining/mm^2^ in the different areas of the atherosclerotic plaque. Magnification 40× (scale bar = 200 µm). (**E**) Representative Western blot images of active caspase 3 (**upper**) and their quantification (**lower**) in transfected HUVECs following serum deprivation (**left**) and transfected VSMCs following thapsigargin exposure (**right**). HUVECs = human umbilical vein endothelial cells; VSMCs = vascular smooth muscle cells; IGF-IIR= insulin-like growth factor type 2 receptor; WT = Wild type group; STD = standard type diet; *ApoE^−/−^* = *ApoE* deficient mice; HFD = high fat diet; IHC = immunohistochemistry; M = media; F = fibrous; S = shoulder; FBS = foetal bovine serum. All the in vitro experiments have been performed at least 3 times (*n* = 3). Western blot of IGF-IIR: WT STD 18 wks (*n* = 6); *ApoE^−/−^* STD 18 wks (*n* = 6); *ApoE^−/−^* HFD 18 wks (*n* = 6). Immunohistochemistry of IGF-IIR: Media (*n* = 44); Fibrous (*n* = 49); Shoulder (*n* = 19).

To study the role of miR-143-3p in the mechanisms involved in atherosclerosis progression, HUVECs and VSMCs were transfected with a specific miRNA precursor to increase miR-143-3p expression. After 72h of transfection, miR-143-3p expression was significantly increased in both HUVECs and VSMCs (Figure 7A). Among its targets, IGF-IIR was selected due to its role in the progression of atherosclerosis (Supplemental Figure S4C,D). 

After 96 h of transfection with the miRNA mimic, IGF-IIR expression was significantly downregulated in HUVECs and VSMCs (Figure 7B). In the same way, HFD-fed *ApoE^−/−^* mice showed lower miR-143-3p expression and a consequent increase in IGF-IIR expression (Figure 1C and Figure 7C, respectively). Moreover, miR-143-3p was downregulated, and in the shoulder regions IGF-IIR was significantly overexpressed in carotid plaque from ACA patients (Figure 2C and Figure 7D).

Since IGF-IIR has been implicated in the instability of advanced atherosclerotic plaques, we performed new experiments to elucidate the relationship between miR-143-3p, IGF-IIR and apoptosis. For that, we induced the activation of caspase 3 by FBS starvation for 6 h in HUVECs or 100 nM thapsigargin treatment for 2 h in VSMCs (Figure 7E). More importantly, when HUVECs and VSMCs were pre-treated with pre-miR-143-3p for 96h the levels of active caspase 3 was significantly reduced in basal conditions or in HUVECs with FBS starvation for 6h (HUVECs) or in VSMCs stimulated with thapsigargin (Figure 7E).

## 3. Discussion

Atherosclerosis has been a silent and asymptomatic disease for decades in which various factors can contribute to the progression and rupture of vulnerable plaques and, consequently, trigger the acute event such as acute myocardial infarction or stroke [21]. In this regard, not only could the study of miRNAs panels help in the future to identify the presence of vulnerable plaques, but also to avoid the progression process. In this study, we have analyzed 18 miRNAs and have finally identified two miRNAs, miR-155-5p and miR-143-3p, with altered levels in both an experimental atherosclerosis model in mice and in patients with advanced carotid atherosclerosis. In this paper, we present a novel role of miR-155-5p and miR-143-3p in the insulin resistance and apoptosis of vascular resident cells, respectively, being both key processes in the progression and instability of atherosclerotic plaque. 

Clearly miR-155 is one of the most dynamically regulated and multifunctional miRNAs, which has been associated with the regulation of immune-related processes, with an impact on cancer [8,13] and atherosclerosis [21]. Diverse studies about the role of miR-155-5p in atherosclerosis have obtained contradictory results and the most plausible explanation might be that the function of miR-155-5p depends on the phase of atherosclerosis. Thus, miR-155-5p could suppress atherosclerosis in the early phases, while promoting its progression in advanced stages [22,23]. For instance, in a model of early atherosclerosis, low-density lipoprotein receptor deficient mice transplanted with miR-155-deficient bone marrow had increased atherosclerotic plaques, elevated levels of pro-inflammatory monocytes, and decreased interleukin (IL)-10 production [24]. However, several studies using *ApoE^−/−^* mice as a model for an advanced phase of atherosclerosis demonstrated that a treatment using antagomiR-155 attenuated atherosclerosis development and progression in *ApoE^−/−^* mice [25]. Similarly, lower plaque sizes were observed in *ApoE^−/−^* mice with a leukocyte specific miR-155 deficiency [26] or in *ApoE^−/−^* miR-155^−/−^ double knockout mice [27]. In accordance with these findings, we observed that miR-155-5p expression significantly increased in aortic roots from *ApoE^−/−^* fed with HFD, a model of advanced atherosclerosis, as well as in carotid biopsies from patients with advanced atherosclerosis. In contrast, subjects with fibrolipidic plaques, an early phase of atherosclerosis, showed lower miR-155-5p levels than healthy subjects. In this regard, previous studies have described that miR-155-5p might be a possible biomarker of plaque instability in the plasma of coronary artery disease (CAD) patients [28] and overexpressed in macrophages during atherosclerosis progression [29], consistent with our findings reporting a noteworthy miR-155-5p overexpression in diabetic patients with advanced carotid atherosclerosis. The miR-155 has also been described to promote inflammatory activation of macrophages by repressing B-cell leukemia/lymphoma (BCL-6), a negative regulator of nuclear factor-kappa B (NF-κB) signaling, thus promoting atherosclerosis [26]. 

Since the role of miR-155-5p in vascular insulin resistance has not been elucidated yet and AKT is a confirmed target of this miRNA [30] that promotes proliferation and survival of VSMCs and ECs during atherosclerosis [30,31,32], we explored whether miR-155-5p could contribute to the development of insulin resistance. We demonstrated that AKT was decreased in the aorta from HFD-fed *ApoE^−/−^* mice and in carotid samples from ACA patients, which correlate with an overexpression of miR-155-5p. However, patients with early atherosclerosis presented a significant increase in AKT protein levels. We also established a significant inverse correlation between miR-155-5p and AKT in human carotid artery. Moreover, in VSMCs and HUVECs a significant decrease in AKT protein levels was noted after miR-155-5p overexpression, and in consequence a strong decrease in AKT phosphorylation and function. Moreover, miR-155-5p also regulates p85α as we observed in VSMCs, which could contribute to the significant decrease in AKT phosphorylation. 

We have also found a significant and robust correlation between BMI and both miR-155-5p and AKT expression. Recently, several miRNAs have emerged as key agents involved in pathways related to obesity such as adipokine expression, glucose and lipid metabolism, insulin signaling, oxidative stress, and inflammation [33,34]. Both the adipose tissue and circulating miRNAs are deregulated in human obesity [35]. Previous studies have described that the expression of miR-155-5p, miR-34a and let-7c might be altered in response to tumor necrosis factor-α (TNF-α) in human adipocytes, and that miR-155-5p is closely associated with NF-κB signalling [36,37]. It has been reported that NF-κB-p65 interacts with the promoter site of miR-155, and thus NF-κB activation promotes miR-155 transcription [38]. In this regard, the overexpression of miR-155-5p observed in advanced atherosclerosis could be induced by NF-κB activation itself since some groups have described a significant NF-κB activation in carotid advanced atherosclerosis [39,40] as well as in aortic root from *ApoE^−/−^* mice under HFD [41], both similar to the samples used by us. 

In this regard, modulation of this inflammation-related miR-155-5p in both adipocytes and their exosomes would improve adipocyte dysfunction and would have an impact on distant organs, such as liver or vascular tissues [42]. Indeed, the deletion of miR-155 in mice prevented diet-induced obesity, improved insulin sensitivity, and abrogated adipocyte hypertrophy and adipose tissue inflammation [43]. Moreover, the reduction in miR-155 increases the expression of genes involved in brown adipogenesis, lipolysis, and energy release, which could synergize to improve fat metabolism [44,45,46,47]. Our results suggest that miR-155-5p might promote the simultaneous development of several pathophysiological processes in different organs, such as vascular and hepatic insulin resistance. Other reports have already described that hepatic miR-155-5p is upregulated in HFD-induced non-alcoholic fatty liver disease (NAFLD) in rats [47] and in non-alcoholic steatohepatitis (NASH) mouse models [48]. Furthermore, miR-155 plays a key role in hepatic lipid metabolism and its deficiency reduces steatosis and fibrosis [49]. Therefore, miR-155-5p coordinately affects various pathways that may elicit adiposity and obesity as well as vascular and hepatic alterations.

The other miRNA candidate that we have studied has been miR-143-3p, which modulates the expression of several genes relevant to cardiovascular biology and function. This miRNA plays a protective role in myocardial infarction by targeting ciclooxygenase-2 [20] or apoptosis-related genes [50]. So, the action of miR-143-3p might be inducing cell migration and inhibiting apoptosis. In this regard, miR-143-3p plays a key role in VSMCs differentiation through the downregulation of its target, Ets-like gene 1, a transcriptional coactivator that is crucial in the regulation of the VSMC phenotype [9]. In our experimental model of advanced atherosclerosis and in patients with ACA, we observed a significant decrease in miR-143-3p whereas in *ApoE^−/−^* mice after only 8 weeks of HFD and in subjects with fibrolipidic plaques, both with early atherosclerosis, miR-143-3p levels did not significantly differ from their respective controls. 

On the other hand, we have also attributed a role to miR-143-3p in the progression of atherosclerosis through the regulation of IGF-IIR. Xihua L et al. confirmed IGF-IIR as a target for miR-143-3p, giving a protective role against atherosclerosis to this miRNA in patients with metabolic syndrome [51]. IGF-IIR is a fetal promoter of cell growth, survival and differentiation [52]. During atherosclerosis, IGF-IIR overexpression may have a protective role in macrophages and a detrimental role in VSMCs or unstable atherosclerotic plaques [53,54]. Moreover, IGF-IIR activates caspase 3 in cardiomyocytes, promoting apoptosis [55]. In our work, we associated a decrease in miR-143-3p with an increase in IGF-IIR expression both in human advanced atherosclerotic plaques and in aortic roots from *ApoE^−/−^* mice fed with HFD. Therefore, miR-143-3p downregulation might have a pro-apoptotic effect in HUVECs and VSMCs. However, further studies are needed to determine the mechanism by which IGF-IIR activates caspase 3 in atherosclerosis.

In summary, this study demonstrates a novel role for miR-155-5p and miR-143-3p in atherosclerosis progression. Our results suggest that miR-155-5p overexpression may be involved in vascular insulin resistance by targeting AKT, whereas miR-143-3p downregulation could be a pro-apoptotic mechanism by increasing IGF-IIR during plaque progression (Graphical Abstract).

## 4. Materials and Methods

### 4.1. Human Samples

We used two cohorts of patients. In the first of them, human aortas were consecutively collected from deceased organ donors from 2010 to 2013 under the authorization of the French Biomedicine Agency (PFS 09-007). After macroscopic examination, the aortas were classified according to Stary classification [56] into two groups: control aortas (CAs, *n* = 7), and aortas with fibrolipidic initial plaques [fibroatheromas (FAs), *n* = 7]. A small portion of tissue from each sample was fixed in 3.7% paraformaldehyde for classical histology and immunochemistry assessments. For the CA samples, it was practically and virtually impossible to separate and independently process the tunica intima. For these samples, the adventitia was carefully removed, and only the results obtained from the tunica media are presented. There were no significant differences in terms of age and gender. The investigation conforms to the principles outlined in the Declaration of Helsinki.

The second cohort corresponds to patients with advanced carotid atherosclerosis. Forty atherosclerotic plaques from patients with carotid stenosis > 70% undergoing carotid endarterectomy at IIS-Fundación Jiménez Díaz (Supplemental Table S3). The plaques showed an increase in inflammatory cells (Stary stages V–VI), whereas adjacent areas were mainly composed of VSMCs and lipid deposits (Stary stage III). The study was approved by the Hospital’s Ethics Committee (IIS-Fundación Jiménez Díaz) with the reference number PI1442016 according to the institutional and the Good Clinical Practice guidelines, which was performed in accordance with the Declaration of Helsinki. All participants gave written informed consent.

### 4.2. Animal Model

Male C57Bl/6 Wild type (WT) and *ApoE* knockout (*ApoE^−/−^*) mice were maintained under standard light (12 h long light/dark cycles), temperature (23.3 °C), and humidity (65.1%) conditions, and ad libitum diet from their weaning, up to their sacrifice. The WT mice (*n* = 7) were fed a standard type diet ([STD] 3% of the kcal are provided by fat, Envigo, USA) for 8 or 18 weeks, while the *ApoE^−/−^* mice were separated in two groups: one was fed the STD (*n* = 7), and the other a HFD (*n* = 10) (60% of the kcal are provided by fat, Envigo, Indianapolis, IN, USA) for 8 or 18 weeks before sacrifice. After 8 or 18 weeks fed with the corresponding diet, animals were sacrificed following fasting for 16 h. All animal experimentation was conducted in accordance with the accepted standards of animal use approved by the Complutense University of Madrid Ethics Committee, Autonomic Community of Madrid (PROEX188/88) and the guidelines from Directive 2010/63/EU of the European Parliament on the protection of animals used for scientific purposes. The microbiological and health state of the mice was controlled by the FELASA (Federation of European Laboratory Animal Science Associations) criteria and showed no pathogenic infection.

For euthanasia, the mice were anesthetized with Ketamine (50 ng/mL, Ketalar^®^ [Pfizer, New York, NY, USA]) and Xylazine (200 mg/mL Rompun^®^ [Bayer, Barmen, Germany]) intraperitoneal injection on a 50:5 dose per Kg. The aorta and the liver were harvested and stored at −80 °C, while the aortic root was washed with saline and then included in Tissue-Tek^®^ O.C.T. Compound ([O.C.T.], VWR BDH Chemicals^®^, Radnor, PA, USA) and stored at −80 °C for further analysis. Both tissues were extracted under sterile conditions. Blood was extracted from the jugular vein and mixed with 0.4% p/v Citrate (Merck, Darmstadt, Germany), then the plasma was recovered after a 1200× *g* centrifuge for 15 min at 4 °C for subsequent analysis. Before the injection, the animals were weighted, and plasma glucose was measured using an Accu-Chek^®^ glucometer (Aviva Roche, Basilea, Switzerland). Finally, cholesterol and triglycerides were tested in plasma samples from fasted mice (Spinreact, Girona, Spain).

In order to study the vascular insulin signaling in physiological conditions, in vivo insulin signaling assays were performed. Fasted mice were intraperitoneally (i.p.) injected with 1 U/kg BW of insulin glulisine (Apidra SoloStar, Sanofi, Paris, France) or an equivalent volume of 0,9% p/v saline solution (*n* = 5 per group). After 10 min, mice were sacrificed, and harvested tissues were immediately frozen in liquid nitrogen. Insulin signaling was assessed by Western blot against phospho-AKT (Ser473) in homogenates of aorta artery.

### 4.3. Cell Culture

HUVECs were purchased from PromoCell. They were grown in MCDB-131 culture medium (Life Technologies, Carlsbad, CA, USA) enriched with L-glutamine 2mM (Gibco^TM^, Fisher Scientific, Hampton, NH, USA), foetal bovine serum 7.5% *v*/*v* (Gibco^TM^, Fisher Scientific, Hampton, NH, USA), Penicillin/Streptomycin 100 U/mL (Gibco^TM^, Fisher Scientific, Hampton, NH, USA) and endothelial growth factor 1X (R&D systems^®^, Minneapolis, MN, USA). Cells were received at passage 2 and were grown until passage 8. All cells were grown at 37 °C in a humified 5% CO_2_ incubator (Fisher Scientific, Hampton, NH, USA). 

Generation of immortalized WT VSMCs lines was previously described [57]. Cell lines were cultured to subconfluence (70–80%) with 10% foetal bovine serum (FBS)-DMEM for in vitro experiments. 

### 4.4. Histological Tissue Samples

Paraffin-embedded human carotids and livers from an experimental model were cut into 5 μm sections and stained with hematoxylin and eosin purchased from PanReac Appli Chem ITW Reagents. AKT and IGF-IIR were detected by immunoperoxidase with rabbit anti-AKT (#9272, Cell Signalling Technology Inc.^®^, Danvers, MA, USA) and anti-IGF-IIR (sc-25462, Santa Cruz Biotechnology, Dallas, TX, USA) polyclonal antibodies. After an overnight incubation with each primary antibody, sections were incubated with a peroxidase-conjugated secondary antibody for 1 h at 1:100 dilution. The sections were stained for 10 min at room temperature with 3,3-diaminobenzidine and then counterstained with hematoxylin and mounted in DPX mounting medium (255,254.1610, PanReac AppliChem ITW Reagent, Sigma-Aldrich, Saint Louis, MO, USA). In each experiment, negative controls without the primary antibody were included to check for nonspecific staining.

The immunohistochemistry images were quantified using the “count and measure objects” tool in the Image-Pro Plus software IPWin (v4.5, Media Cybernetics, Rockville, USA). The color considered as positive staining for the same protein was manually selected, and the value corresponding to the sum of all stained areas was obtained. The results were expressed as the percentage of the stained area with respect to the total area analyzed in each sample. 

Hepatic H&E staining was performed in paraffin-embedded sections (4 µm thick) that were evaluated by a single-blinded highly qualified hepatopathologist from Santa Cristina Hospital (Madrid, Spain). The score range was set between 0 and 3. Steatosis score was assessed, grading percentage involvement by steatotic hepatocytes as follows: grade 0, 0–5%; grade 1, >5–33%; grade 2, >33–66%; grade 3, >66%.

Aortic root and a section of liver samples was included in Tissue-Tek^®^ optimum cutting temperature (OCT) compound (Sakura Finetek, Alphen aan den Rijn, The Netherlands), and later in liquid nitrogen for freezing. The O.C.T. embedded aortic root or liver samples from the experimental model were cut into 5 μm sections using a cryostat (CM1510 S; Leica, Wetzlar, Germany). Cut sections were stained with Oil Red O and hematoxylin to assess lipid depot. Individual lesion area in aortic roots was determined by averaging the maximal values. A stock Oil Red O (Sigma-Aldrich, USA) solution was made with 3 mg/mL Oil Red O in 99% isopropanol. The stock was diluted in a 3:2 ratio in ultrapure water. Frozen cryosections were air dried, fixed in 10% formalin, stained with Oil Red O and counterstained with Mayer’s hematoxylin (Electron Microscopy Sciences, Hatfield, PA, USA). The sections were mounted with aqueous mounting medium for imaging (Vector Labs, Newark, CA, USA). Images of sections were acquired using an inverted Eclipse TE300 microscope coupled to a Digital Sight DS-U2 camera (Nikon, Tokyo, Japan). Quantifications for images of Oil Red O staining in aortic roots were performed using IP Win32 v4.5 software (Acromag, Wixom, MI, USA). Finally, % stenosis and % lesion area/total area was analyzed using Image J (v1.52a, Wayne Rasband, National Institute of Health, Stapleton, NY, USA).

### 4.5. En Face Imaging of Aorta

Atherosclerotic lesions were quantified by *en face* analysis of the whole aorta. For *en face* preparations, the aorta was opened longitudinally, while still attached to the heart and major branching arteries in the body. The aorta from the heart to the iliac bifurcation was then removed and was pinned out on a white wax surface in a dissecting pan using stainless steel pins 0.2 mm in diameter. After overnight fixation with 4% paraformaldehyde and PBS rinsing, the aortas were stained for 6 min in a filtered solution containing 0.5% Oil Red-O, 35% ethanol and 50% acetone, and then destained in 80% ethanol. The Oil Red-O-stained aortas were photographed, and the atherosclerotic lesions were quantified using IP Win32 v4.5 software.

### 4.6. miRNA Extraction from the Aorta, Liver, Vascular Cell Lines and Paraffin-Embedded Carotid Tissue

The miRNA content from the aorta, the liver and the cells were extracted following the mirVana^TM^ miRNA Isolation Kit (Invitrogen^TM^, Thermo Fisher Scientific, Waltham, MA, USA). The miRNA content from paraffin-embedded carotids was extracted using the RNeasy FFPE kit (Qiagen, Hilden, Germany). All the extractions were made following the protocol handled by the manufacturers. In all cases, miRNAs and long RNAs were obtained in separate fractions. The miRNA sample concentration was then determined using a NanoDrop^TM^ 2000 and the NanoDrop 2000/2000c Operating Software (Thermo Scientific, Waltham, MA, USA). 

### 4.7. Cell Transfection with miRNA Precursors

Precursors of miR-155-5p and miR-143-3p were purchased from Sigma-Aldrich. Approximately 5 × 10^4^ cells were seeded in P60 culture plates (353002, Falcon^TM^, Thermo Fisher Scientific, Waltham, MA, USA) and transfected with 10–20 nM of MISSION^®^ miRNA mimic hsa-miR143-3p or hsa-miR-155-5p (HMI0221 or HMN0254, Sigma-Aldrich, Saint Louis, MO, USA). As specified in the manufacturer’s protocol (#409-10, Polyplus transfection^®^, Strasbourg, France), miRNA expression in transfected cells was assessed 72 h after transfection, whereas protein downregulation was analysed 96 h following transfection. To evaluate insulin signaling in cells transfected with pre-miR-155-5p, they were deprived in 0% FBS medium for 6h and then stimulated with 100 nM insulin (Sigma-Aldrich, Saint Louis, MO, USA) for 10 min. To study the effect of the pre-miR-143-3p on cell apoptosis, HUVECs were deprived of FBS for 6 h or VSMCs were deprived of FBS for 2 h followed by a treatment with thapsigargin for 2 h (100 nM, Santa Cruz Biotechnology, Dallas, TX, USA). 

### 4.8. RT-qPCR Analysis

Complementary DNA (cDNA) was synthesized by a High-Capacity cDNA Reverse Transcription Kit (Applied Biosystems, Foster City, CA, USA) for mRNA analysis. Quantitative polymerase chain reaction (qPCR) was done using cDNA as template and the TaqMan^®^ Fast Advanced Master Mix (Thermo Scientific, Waltham, MA, USA). The genes were detected using TaqMan^®^ (Thermo Scientific, Waltham, MA, USA) probes for hsa-miR-143-3p (477912_mir, mature miRNA sequence: UGAGAUGAAGCACUGUAGCUC), mmu-miR-155-5p (mmu480953_mir, mature miRNA sequence: UUAAUGCUAAUUGUGAUAGGGGU) and mmu-miR-191-5p (mmu481584_mir, mature miRNA sequence: CAACGGAAUCCCAAAAGCAGCUG) used as endogenous gene. The references of other Taq-Man^®^ probes used are indicated in the Supplemental Table S2. All the probes detect both mouse and human target genes. All RT-qPCR experiments were performed in an ABI Prism 7900HT Thermal Cycler (Applied Biosystems, Foster City, CA, USA).

The relative abundance of mRNA targets, normalized with the endogenous gene and relative to the control, is calculated as follow a: Relative Quantification (RQ) = 2^−ΔΔCt^; ΔCt (cycle threshold) = Ct (miRNA target) − Ct (miR-191-5p); ΔΔCt = ΔCt for any sample − ΔCt for the control]. Amplification of miR-191-5p was used in the same reaction of all samples as an internal control.

### 4.9. Western Blot Analysis

Proteins from cell lysates (20–60 μg), and tissue samples (20–70 µg) were separated on a 10% or 10–20% gradient acrylamide gel and then transferred to a 0.45 μM pore PVDF membrane (Merck, Darmstadt, Germany) as previously described [57]. The primary antibodies used are shown in Supplemental Table S4 and all of them were diluted in TTBS. Rabbit and mouse primary antibodies were immunodetected using horseradish peroxidase-conjugated anti-rabbit IgG (NA931V; 1:4000 in TTBS) or anti-mouse IgG secondary antibody (NA934V; 1:5000 in TTBS) (GE Healthcare, Buckinghamshire, UK), respectively. When possible, phospho-proteins and their total expression were detected in the same gel, using Restore^TM^ Western Blot Stripping Buffer (Thermo Fisher Scientific) as per the manufacturer’s instructions, blocking the membrane again before the incubation with the next antibody. Loading was normalized by β-actin or α-tubulin. Protein bands were visualized using the Clarity Western Blot Analysis ECL (BioRad^®^, Hercules, CA, USA). Band densitometry was analysed using ImageJ Software (v1.52a, Wayne Rasband, National Institute of Health, Stapleton, NY, USA).

### 4.10. Database Search to Find miRNAs and Their Possible Targets

The miRNAs analyzed in the study were identified by an exhaustive search for keywords (miRNAs, atherosclerosis, insulin resistance, inflammation, and fatty liver) and different publications in PubMed. Once the miRNAs of interest were selected, the interaction between them and their mRNA targets was evaluated in miRNA-Target Interaction databases such as GEO Database, TargetScan, miRWalk, miRDB, DIANA and miRTarBase. The data collected from these databases were analyzed, and only those targets that appeared in two or more databases were considered as possible targets. A diagram showing the proposed regulatory axes during atherosclerosis progression is supplied in the Supplemental Figure S4B,D. These representations were made using Cytoscape software (v.3.8.2. on Java 11.0.6. by AdoptOpenJDK, Darmstadt, Germany).

### 4.11. Statistical Analysis

The data from the experimental groups were analyzed using GraphPad Prism (v8, JPM ®, New York, USA). Normality and Lognormality tests were performed to confirm that the data followed a normal distribution. Statistical significance of the differences between groups was assessed by Student’s t tests when comparing two groups, or with ANOVA tests followed by a Bonferroni *post-hoc* test when comparing more than two groups. Correlation between variables was assessed by two-tailed Spearman’s r correlation analyses. The exact *p* value is indicated in each figure when it reached statistically significance (*p* < 0.05). 

## Data Availability

The datasets used and/or analyzed during the current study are available from the corresponding authors (algomezh@ucm.es or oescriba@ucm.es) on reasonable request.

## Supplementary Materials

The following supporting information can be downloaded at: https://www.mdpi.com/article/10.3390/ijms231810253/s1.
