# Supplementary material for: Implication of miR-155-5p and miR-143-3p in the Vascular Insulin Resistance and Instability of Human and Experimental Atherosclerotic Plaque
Source: Int J Mol Sci. 2022 Sep 6;23(18):10253. doi: 10.3390/ijms231810253 (PMC9499612; doi:10.3390/ijms231810253)
Supplement: Supplementary file 1 [file ijms-23-10253-s001.zip › ijms-1783757-supplementary.v2.pdf]

SUPPLEMENTAL DATA

SUPPLEMENTAL FIGURE LEGENDS

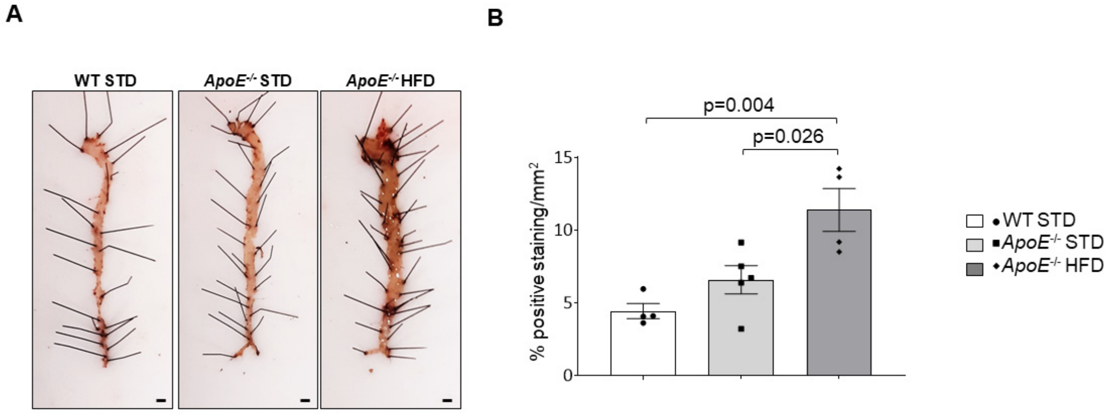

Supplemental Figure S1. Analysis of atherosclerosis in the aorta artery of experimental mouse model. *En face* staining of aortas from male C57BL/6 mice fed a standard diet, male ApoE$^{-/-}$ mice fed a STD and male *ApoE*$^{-/-}$ mice fed a HFD. Scale bar = 100 µm**.** WT= Wild type group; STD= standard type diet; *ApoE*$^{-/-}$ = *ApoE* deficient mice; HFD= high fat diet. WT STD 18wks (n=4); *ApoE*$^{-/-}$ STD 18wks (n=5); *ApoE*$^{-/-}$ HFD 18wks (n=4).

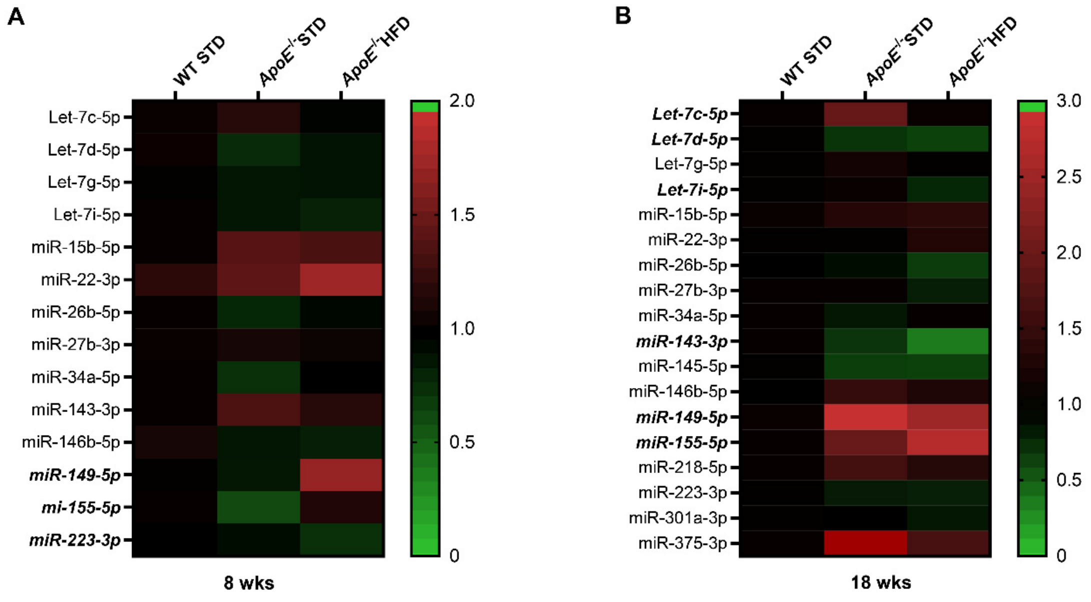

Supplemental Figure S2. Heatmaps of miRNA studied in the aorta artery from experimental model of atherosclerosis. By qRT-PCR, we analyzed 14 and 18 miRNAs in thoracic aorta arteries from WT, ApoE$^{-/-}$ STD and ApoE$^{-/-}$ HFD at 8 **(A)** and 18 **(B)** weeks of diet, respectively. With the mean values of RQ (0-3) of each group, we performed two heatmap using GraphPad Prism v8. The p values are indicated in the Supplemental Table 4.

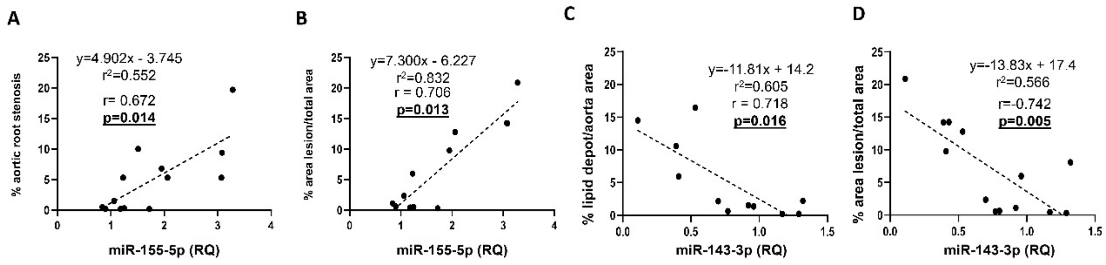

Supplemental Figure S3. Correlation between miR-155-5p and miR-143-3p and atherosclerosis progression. By Spearman´s correlation, we set different correlation between miR-155-5p and % aortic root stenosis **(A)** or % area lesson/total area **(B)**; and miR-143-3p and % lipid depot/aorta area **(C)** or % area lesson/total area **(D)**.

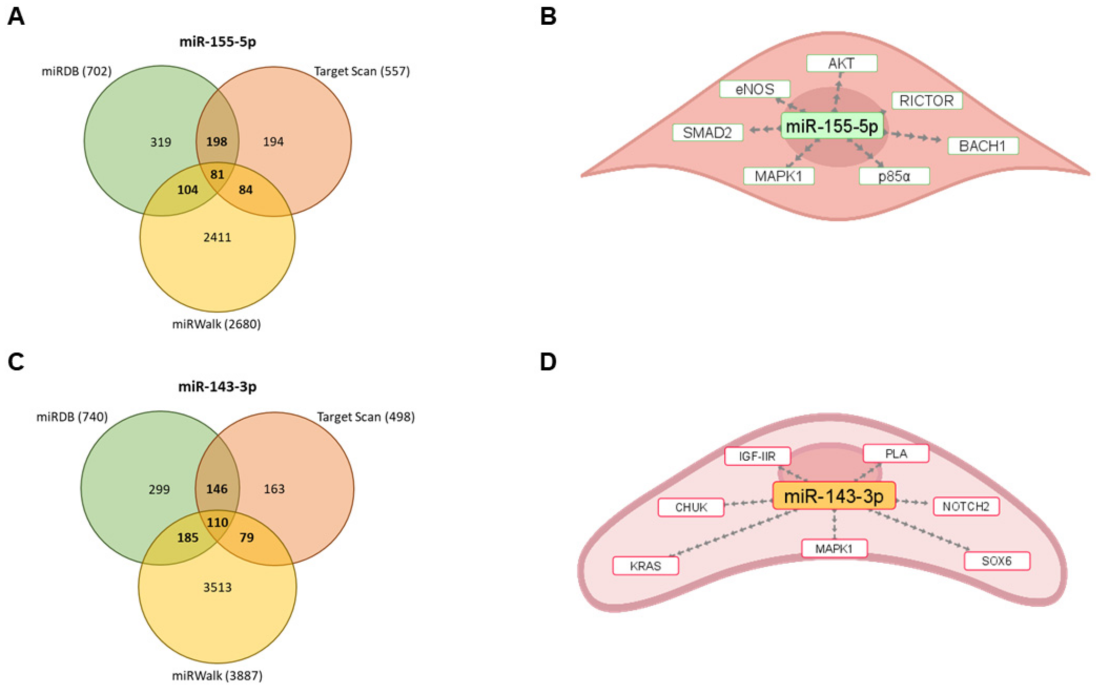

Supplemental Figure S4. Possible targets stood out after *in silico* analysis for both miR-155-5p and miR-143-3p. Venn diagram that represents the different results obtained for miR-155-5p **(A)** or miR-143-3p **(B)** in databases like miRDB, TargetScan and miRWalk. Graphics that show some possible targets for miR-155-5p **(C)** and miR-143-3p **(D).**

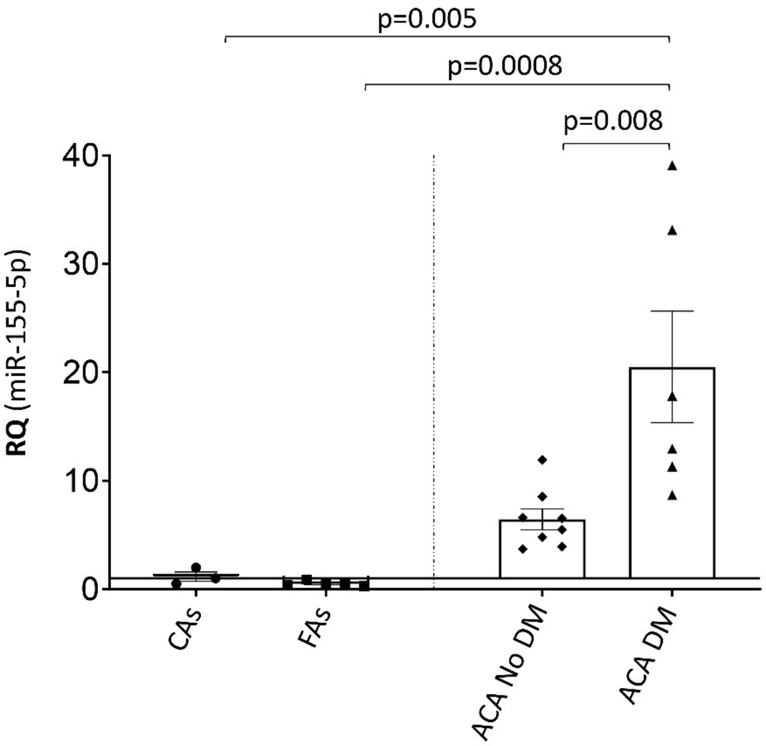

Supplemental Figure S5. Analysis of miR-155-5p in atherosclerotic diabetic and non-diabetic patients. The ACA patients were separated having into account if they have been diagnosed with diabetes or not. miR-155-5p expression was analysed by qPCR. FAs=fibrolipidic plaque; ACA= advanced carotid atherosclerotic plaque. Controls (n=3); fibrolipidic (n=5); ACA (non-diabetic) (n=8); ACA (diabetic) (n=6).

SUPPLEMENTAL TABLES

Supplemental Table S1. Characterization of the mouse experimental model

	**WT STD 8 wks**	***ApoE$^{-/-}$* STD 8 wks**	***ApoE$^{-/-}$* HFD 8 wks**	**WT STD 18 wks**	***ApoE$^{-/-}$* STD 18 wks**	***ApoE$^{-/-}$* HFD 18 wks**
**Weight (g)**	25,9±0,3	26,6±0,5	30,6±0,7 (****, ####)	28,5±0,3	30,1±0,4	35,8±1,2 (****, ####)
**Weight gain (g)**	9,8±0,5	12,9±0,6 (***)	15,3±0,5 (****, ##)	12,8±0,6	15,8±0,6 (**)	21,0±0,9 (****, ####)
**Cholesterol (mg/dL)**	96,4±6,5	188,9±16,8 (***)	209,8±2,1 (****)	117,5±3,9	201,9±8,3 (****)	234,0±6,5 (****, ##)
**Triglycerides (mg/dL)**	49,2±4,9	84,8±9,5 (*)	88,5±6,7 (**)	30,7±3,1	72,8±8,1 (***)	86,4±5,4 (****)

This table collects the weight (g), weight gain (g), cholesterol (mg/dL) and triglycerides (mg/dL) of the six mouse groups: WT fed with STD, and *ApoE$^{-/-}$* fed with STD and HFD at 8 and 18 weeks. Statistical significance between the different groups of 8 weeks or 18 weeks, respectively, was assessed with an ordinary one-way ANOVA. *p<0.05 vs WT STD; **p<0.01 vs. WT STD; ***p<0.001 vs. WT STD; ****p<0.0001 vs. WT STD; ##p<0.01 vs. *ApoE$^{-/-}$* STD, ####p<0.0001 vs *ApoE$^{-/-}$*STD

Supplemental Table S2. miRNAs studied in experimental atherosclerosis model and human atherosclerosis samples. Upregulation or downregulation is only indicated when the p value is statistically significant (p<0.05). WT STD 8 weeks (n=6-7); *ApoE*$^{-/-}$ STD 8 weeks (n=4-5); *ApoE*$^{-/-}$ HFD 8 weeks (n=7-8); WT STD 18 weeks (n=5-7); *ApoE*$^{-/-}$ STD 18 weeks (n=6-8); *ApoE*$^{-/-}$ HFD 18 weeks (n=7-8); CAs (n =5); FAs (n=6); ACA (n=9-12).

		**8 weeks-fed mouse model**		**18 weeks-fed mouse model**		**Human samples**	
**miRNAs**	**Reference**	**Change**	**p value**	**Change**	**p value**	**Change**	**p value**
**hsa-let-7c-5p**	478577_mir	None (*ApoE*$^{-/-}$ STD vs. WT)<br>None (*ApoE*$^{-/-}$ HFD vs. WT)	>0.999<br>>0.999	**Upregulation** (*ApoE*$^{-/-}$ STD vs. WT)<br>**Downregulation** (*ApoE*$^{-/-}$ HFD vs. STD)	**0.035**<br>**0.030**	None (ACA vs. CAs)<br>None (ACA vs. FAs)	0.230<br>0.332
**hsa-let-7d-5p**	478439_mir	None (*ApoE*$^{-/-}$ STD vs. WT)<br>None (*ApoE*$^{-/-}$ HFD vs. WT)	0.616<br>>0.999	None (*ApoE*$^{-/-}$ STD vs. WT)<br>**Downregulation** (*ApoE*$^{-/-}$ HFD vs. WT)	0.081<br>**0.027**	None (ACA vs. CAs)<br>None (ACA vs. FAs)	0.753<br>0.641
**hsa-let-7g-5p**	478580_mir	None (*ApoE*$^{-/-}$ STD vs. WT)<br>None (*ApoE*$^{-/-}$ HFD vs. WT)	>0.999<br>>0.999	None (*ApoE*$^{-/-}$ STD vs. WT)<br>None (*ApoE*$^{-/-}$ HFD vs. WT)	0.570<br>>0.999		
**hsa-let-7i-5p**	478375_mir	None (*ApoE*$^{-/-}$ STD vs. WT)<br>None (*ApoE*$^{-/-}$ HFD vs. WT)	>0.999<br>0.450	None (*ApoE*$^{-/-}$ STD vs. WT)<br>**Downregulation** (*ApoE*$^{-/-}$ HFD vs. STD)	>0.999<br>**0.021**	None (ACA vs. CAs)<br>None (ACA vs. FAs)	>0.999<br>>0.999
**mmu-miR-15b-5p**	mmu482957_mir	None (*ApoE*$^{-/-}$ STD vs. WT)<br>None (*ApoE*$^{-/-}$ HFD vs. WT)	0.561<br>0.640	None (*ApoE*$^{-/-}$ STD vs. WT)<br>None (*ApoE*$^{-/-}$ HFD vs. WT)	>0.999<br>>0.999		
**mmu-miR-22-3p**	mmu481004_mir	None (*ApoE*$^{-/-}$ STD vs. WT)<br>None (*ApoE*$^{-/-}$ HFD vs. WT)	>0.999<br>0.355	None (*ApoE*$^{-/-}$ STD vs. WT)<br>None (*ApoE*$^{-/-}$ HFD vs. WT)	>0.999<br>0.697		
**mmu-miR-26b-5p**	mmu482965_mir	None (*ApoE*$^{-/-}$ STD vs. WT)<br>None (*ApoE*$^{-/-}$ HFD vs. WT)	0.728<br>>0.999	None (*ApoE*$^{-/-}$ STD vs. WT)<br>None (*ApoE*$^{-/-}$ HFD vs. WT)	>0.999<br>0.056	None (ACA vs. CAs)<br>None (ACA vs. FAs)	>0.999<br>>0.999
**mmu-miR-27b-3p**	mmu478270_mir	None (*ApoE*$^{-/-}$ STD vs. WT)<br>None (*ApoE*$^{-/-}$ HFD vs. WT)	>0.999<br>>0.999	None (*ApoE*$^{-/-}$ STD vs. WT)<br>None (*ApoE*$^{-/-}$ HFD vs. WT)	>0.999<br>>0.999		
**mmu-miR-34a-5p**	mmu481304_mir	None (*ApoE*$^{-/-}$ STD vs. WT)<br>None (*ApoE*$^{-/-}$ HFD vs. WT)	0.459<br>>0.999	None (*ApoE*$^{-/-}$ STD vs. WT)<br>None (*ApoE*$^{-/-}$ HFD vs. WT)	>0.999<br>>0.999		
**hsa-miR-145-5p**	477916_mir			None (*ApoE*$^{-/-}$ STD vs. WT)<br>None (*ApoE*$^{-/-}$ HFD vs. WT)	0.274<br>0.248		
**mmu-miR-146b-5p**	mmu478513_mir	None (*ApoE*$^{-/-}$ STD vs. WT)<br>None (*ApoE*$^{-/-}$ HFD vs. WT)	>0.999<br>>0.999	None (*ApoE*$^{-/-}$ STD vs. WT)<br>None (*ApoE*$^{-/-}$ HFD vs. WT)	0.255<br>0.784		
**mmu-miR-149-5p**	mmu480946_mir	None (*ApoE*$^{-/-}$ STD vs. WT)<br>**Upregulation** (*ApoE*$^{-/-}$ HFD vs. STD)	>0.999<br>**0.043**	**Upregulation** (*ApoE*$^{-/-}$ STD vs. WT)<br>None (*ApoE*$^{-/-}$ HFD vs. WT)	**0.015**<br>0.057	None (ACA vs. CAs)<br>Undetermined data for FAs	0.317
**mmu-miR-218-5p**	mmu481001_mir			None (*ApoE*$^{-/-}$ STD vs. WT)<br>None (*ApoE*$^{-/-}$ HFD vs. WT)	0.243<br>0.976		
**mmu-miR-223-3p**	mmu481007_mir	None (*ApoE*$^{-/-}$ STD vs. WT)<br>**Downregulation** (*ApoE*$^{-/-}$ HFD vs. WT)	>0.999<br>**0.022**	None (*ApoE*$^{-/-}$ STD vs. WT)<br>None (*ApoE*$^{-/-}$ HFD vs. WT)	0.600<br>0.460		
**hsa-miR-301a-3p**	477815_mir			None (*ApoE*$^{-/-}$ STD vs. WT)<br>None (*ApoE*$^{-/-}$ HFD vs. WT)	>0.999<br>0.625		
**mmu-miR-375-3p**	mmu481141_mir			None (*ApoE*$^{-/-}$ STD vs. WT)<br>None (*ApoE*$^{-/-}$ HFD vs. WT)	0.079<br>>0.999		

Supplemental Table S3: Clinical characteristics of patients bearing advanced carotid atherosclerosis

	**ACA Patients (n=14)**
**Age, years**	70 ± 7
**Gender (male/female), %**	71,4% / 28,6%
**BMI (Kg/m^2)**	29,2 ± 6,3
**Diabetes mellitus, %**	42,9%
**Hypertension, %**	85,7%
**Coronary artery disease, %**	100%
**Current smoking, %**	35,70%

Supplemental Table S4. Primary antibodies used

Antibody	Dilution	Supplier	Reference	Technique
**IGF-IIR (H-300)**	1:250	Santa Cruz Biotechnology	sc-25462	WB
	1:50			IHC
**p-AKT (S473) (193H12)**	1:1000	Cell Signalling Technology Inc	#4058	WB
**AKT**	1:1000	Cell Signalling Technology Inc	#9272	WB
	1:100			IHC
**eNOS**	1:1000	Merck Millipore	#07-520	WB
**p85α (EPR18702)**	1:1000	Abcam	Ab191606	WB
**Cleaved Caspase 3 (D175)**	1:1000	Cell Signalling Technology Inc	#9661	WB
**β-Actin**	1:5000	Sigma Aldrich	A5441	WB

WB: Western-blot; IHC: Immunohistochemistry.
